# Post‐mortem biopsy of a patient with late exacerbation of COVID‐19 pneumonia

**DOI:** 10.1002/rcr2.724

**Published:** 2021-02-22

**Authors:** Kuniko Takahashi, Koichiro Kajiura, Michitaka Nasu, Kei Nakamura, Kazuki Sugata, Akiko Matsuzaki

**Affiliations:** ^1^ Department of Emergency and Critical Care Medicine Urasoe General Hospital Okinawa Japan; ^2^ Department of Thoracic Center Urasoe General Hospital Okinawa Japan; ^3^ Department of Pathology Urasoe General Hospital Okinawa Japan

**Keywords:** COVID‐19, late exacerbation, PCR, post‐mortem biopsy

## Abstract

Pathological findings of coronavirus disease (COVID‐19) have rarely been reported owing to its contagious nature. Here, we treated an 82‐year‐old man whose condition was diagnosed as COVID‐19 pneumonia, which exacerbated approximately 25 days after the initial onset. The patient died despite receiving intensive care. Post‐mortem percutaneous needle biopsy of the lungs and liver tissue was performed, including genomic analysis, immunochemical tests, and pathological studies. Histopathology of the lungs showed both exudative and organizing diffuse alveolar damage. Supposedly, the organizing phase of acute respiratory distress syndrome (ARDS) induced COVID‐19. Polymerase chain reaction (PCR) test and immunostaining of biopsy specimens showed negative results for COVID‐19. Post‐mortem percutaneous needle biopsy was more effective in reducing the risk of contagiousness than autopsy.

## Graphical abstract

Here, we describe pathological findings detected through post‐mortem percutaneous needle biopsy of a patient with late‐onset severe pneumonia from coronavirus disease (COVID‐19).

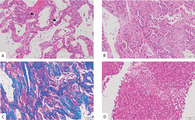

## Introduction

Late in December 2019, an outbreak of infections caused by a novel coronavirus named severe acute respiratory syndrome coronavirus 2 (SARS‐CoV‐2) was reported in Wuhan, Hubei Province, China [1]. The World Health Organization named the SARS‐CoV‐2 infection as coronavirus disease (COVID‐19). COVID‐19 instantly spread worldwide, thereby becoming a pandemic. The clinical features of COVID‐19 are diverse, ranging from asymptomatic to severe pneumonia and death [2]. Genome sequencing of the virus and development of therapeutic agents and vaccines have been researched worldwide; however, pathological findings of this disease have been scarcely reported because of its contagious nature (in humans) [3]. Here, we describe pathological findings detected through post‐mortem percutaneous needle biopsy of a patient with late‐onset severe pneumonia from COVID‐19.

## Case Report

**Figure 1:**
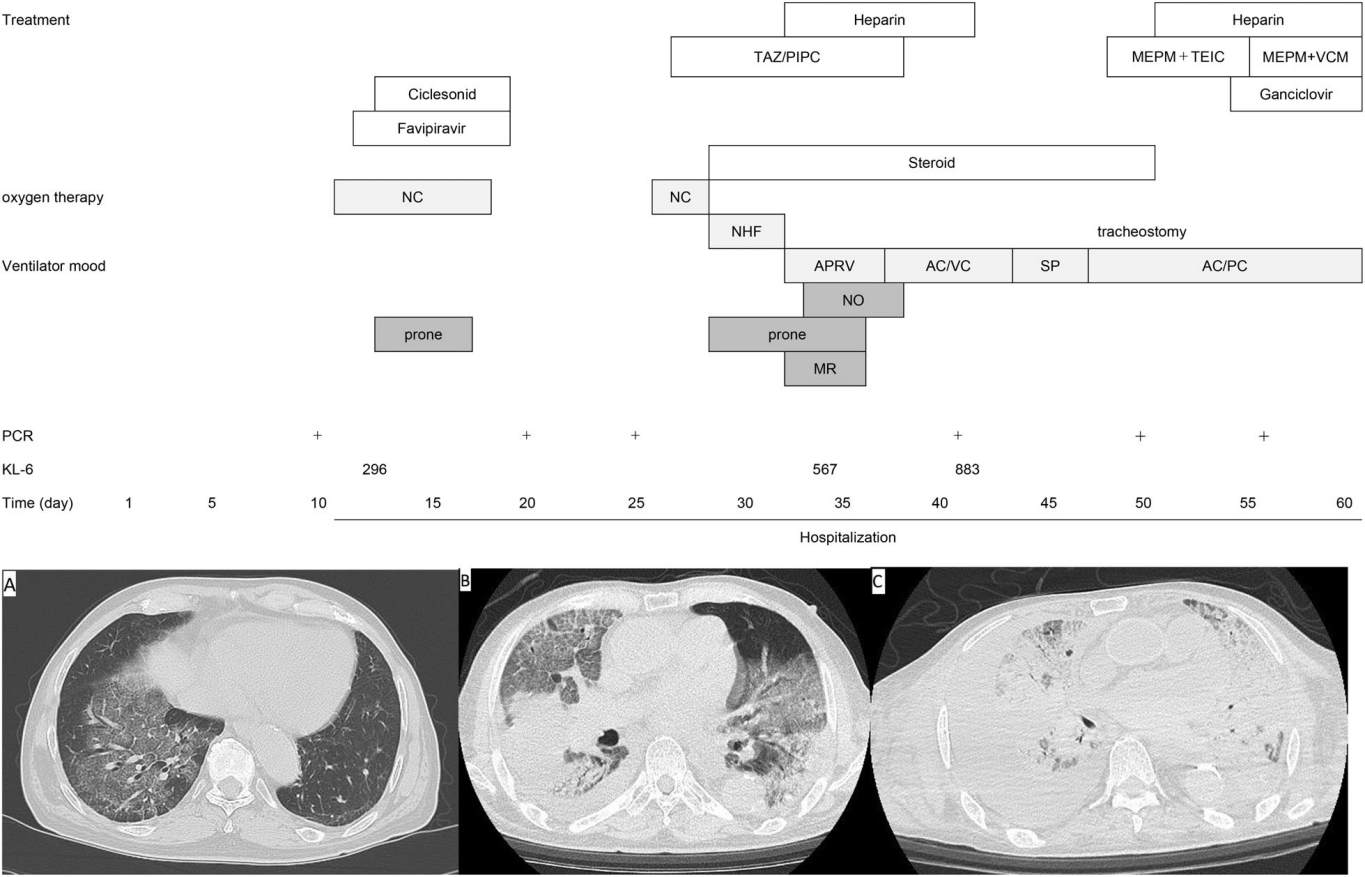
Summary of treatment progress. Chest CT images of the patient at different stages of COVID‐19. (A) Axial view of CT from the previous hospital (day 8) shows GGO predominantly spread on the right lower lobe. (B) Axial view of CT on intubation (day 33) shows GGO spread to bilaterally and consolidation on the right lower lobe. (C) Axial view of CT on post‐mortem autopsy imaging (day 60) shows bilateral spread of consolidation and bilateral pleural effusion. AC/PC, assist control/pressure control; AC/VC, assist control/volume control; APRV, airway pressure release ventilation; COVID‐19, coronavirus disease; CT, computed tomography; GGO, ground‐glass opacity; MPEM, meropenem; MR, muscle relaxant; NC, nasal cannula; NHF, nasal high flow; NO, nitric oxide; SP, spontaneous ventilation; TAZ/PIPC, tazobactam/piperacillin; TEIC, teicoplanin.

The patient was an 82‐year‐old man who presented with low‐grade fever and dry cough at another hospital, where he tested positive for COVID‐19 through a polymerase chain reaction (PCR) test. He was referred to our hospital with symptoms of COVID‐19 pneumonia. He had a history of diabetes and hypertension but did not smoke. Furthermore, he had been administered a calcium channel blocker and an angiotensin‐converting enzyme (ACE) inhibitor. Chest computed tomography (CT) revealed multicentric peripheral ground‐glass opacity (GGO), especially in the right lower lobe (Fig. 1A).

A summary of the treatment protocol is shown in Figure 1. We administered favipiravir and ciclesonide, alongside oxygen therapy. On day 18, peripheral oxygen saturation (SpO_2_) and dyspnoea improved, his fever subsided, and the oxygenation therapy was withdrawn. However, the PCR tests remained positive from days 20 to 25. On day 26, SpO_2_ worsened, his fever returned, and oxygen therapy was resumed. Although the culture test results were negative, antibiotics (tazobactam/piperacillin, TAZ/PIPC) were administrated empirically for suspected complications of secondary infection. The patient needed intubation on day 33. CT revealed consolidation of the right lower lobe around the GGO (Fig. 1B).

The patient received mechanical ventilation in airway pressure release ventilation mode. This was in addition to nitric oxide (NO) inhalation and methylprednisolone (60 mg/day) administration to counter acute respiratory distress syndrome (ARDS). Furthermore, we administered a muscle relaxant along with fentanyl and midazolam for sedation. The patient's condition worsened on day 42, despite providing intensive care. At this stage, blood culture revealed the presence of *Escherichia coli*, and *Stenotrophomonas maltophilia* was detected in the sputum culture, suggesting ventilation‐associated pneumonia. The patient died on day 60. Autopsy CT revealed bilateral lung consolidation along with bilateral pleural effusion (Fig. 1C). There was no obvious abscess in any organ.

**Figure 2:**
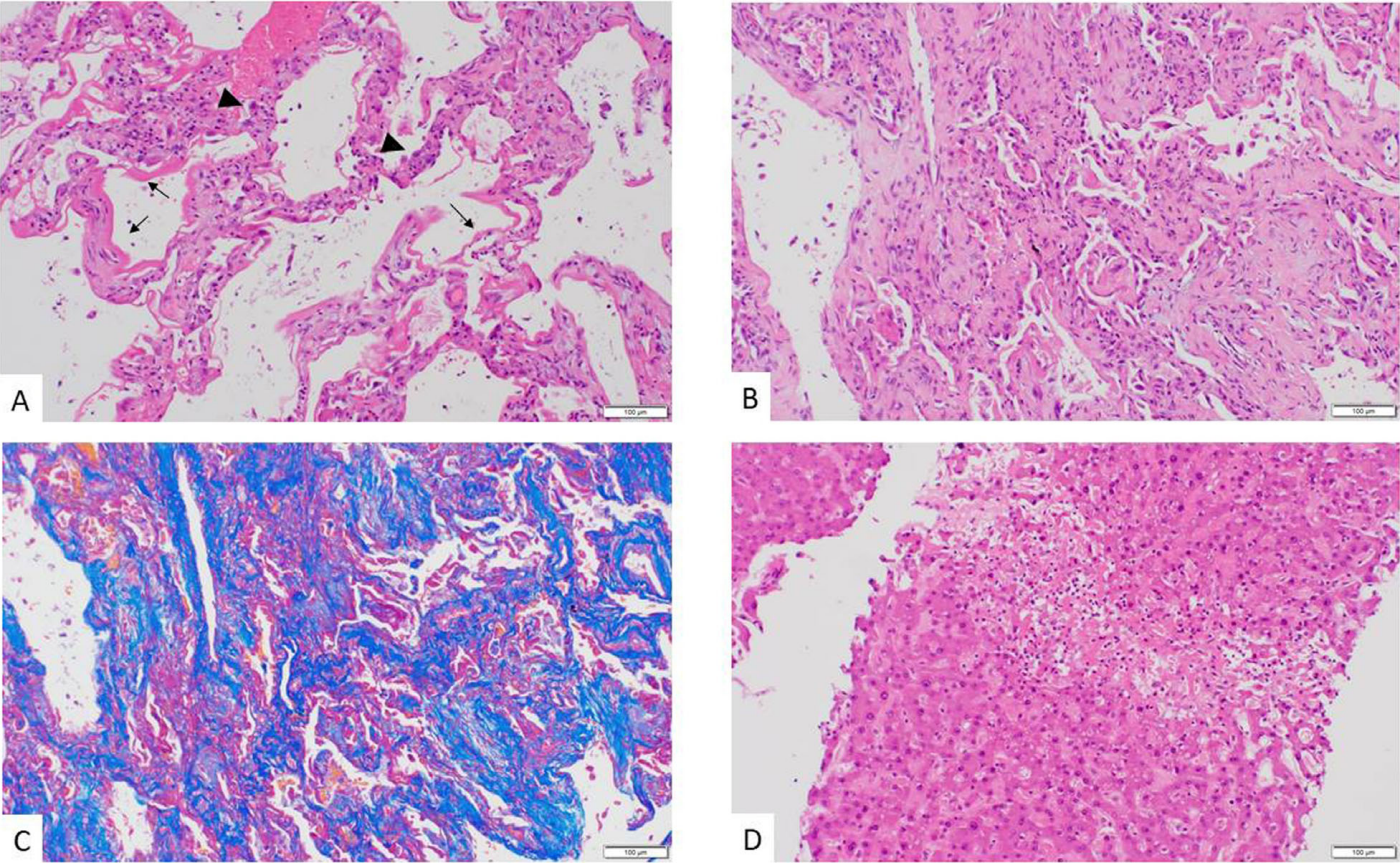
Histopathology of the patient's post‐mortem percutaneous needle lung biopsy sample. (A) Diffuse alveolar damage (DAD) with hyaline membranes (→), intra‐alveolar haemorrhage, and reactive type II pneumocytes (▸) (haematoxylin and eosin (H&E) stain). (B) Intra‐alveolar fibrosis (H&E stain). (C) Intra‐alveolar fibrosis with azan stain. (D) Liver micro‐abscess. Scale bar: 100 μm on each image.

After obtaining informed consent from his family, we performed post‐mortem percutaneous needle biopsies of the lungs and liver tissues with a 14‐G needle. Histopathology of the lungs showed both exudative and organizing diffuse alveolar damage (DAD) (Fig. 2). In the exudative phase of DAD, the lung tissues exhibited prominent hyaline membranes with minimal chronic inflammation, while those in the organizing phase showed intra‐alveolar fibrosis. In addition, they revealed intra‐alveolar haemorrhage and reactive type II pneumocytes. Vasculitis, thrombosis, and the other pathogens were absent. There was no obvious difference between the right and left lungs. In the liver, several micro‐abscesses due to sepsis were detected. Real‐time reverse transcription PCR and immunostaining of these biopsy specimens for COVID‐19 were performed by the Department of Pathology, National Institute of Infectious Disease in Japan [4], all of which were negative. On the basis of the above‐mentioned histopathological and genomic analyses, we considered that the cause of death was ARDS due to cytokine storm, and immune responses, rather than secondary infection or other factors, induced COVID‐19.

## Discussion

Some autopsy reports of COVID‐19 patients have revealed certain clinicopathological features [3]. Autopsies could help evaluate genomic analysis results and immunochemical findings considering the pathological findings of each organ. However, performing an autopsy carries a risk of infection for pathologists and assistants. In this case, post‐mortem percutaneous needle biopsies, including pathological studies, genomic analysis, and immunochemical tests, were performed on specimens. In addition, it was possible to choose the organ from which tissue could be obtained for testing, thus reducing the risk of staff infection. Therefore, post‐mortem percutaneous needle biopsy could be an effective method to determine the cause of death in COVID‐19 patients.

In this case, genomic and immunochemical test results of the lungs and liver were negative for COVID‐19. However, PCR conducted using throat swabs or sputum showed a positive result multiple times. Among the molecular mechanisms of disease exacerbation, which contribute to cytokine storm and immune responses in COVID‐19 patients, the ACE 2 receptor‐mediated inflammatory response plays an important role [5]. In this case, the exacerbation of COVID‐19 might have been because of this mechanism rather than the direct infection of the lung by COVID‐19.

Generally, the reported median time from onset of symptoms to ARDS or admission to the intensive care unit (ICU) is 9.0–10.5 days in COVID‐19 patients [1]. In this case, the patient's COVID‐19 pneumonia was considered to have exacerbated late because his condition worsened about 25 days after the onset of symptoms with persistent positive PCR test results. There have been a few reports regarding the late exacerbation of COVID‐19 pneumonia, and the mechanism underlying it remains unknown. To date, late exacerbation of COVID‐19 was considered rare, and it was implicated that COVID‐19 patients only need a certain period (about one to two weeks) of isolation and observation. However, in this case, the patient exacerbated 25 days after the initial onset, even under proper medication and care. Therefore, more information is required for better management of exacerbated cases of COVID‐19.

### Disclosure Statement

Appropriate written informed consent was obtained for publication of this case report and accompanying images.
